# Muscle-sparing aortic coarctation repair

**DOI:** 10.1016/j.xjtc.2020.05.005

**Published:** 2020-05-17

**Authors:** Stephanie G. Berset, Hitendu Dave, Christian Balmer, Anna Nowacka, Raymond Pfister, Patrick O. Myers, René Prêtre

**Affiliations:** a Department of Internal Medicine, Vaud University Hospital, Lausanne, Switzerland; b Department of Cardiology, Zurich University Children's Hospital, Zurich, Switzerland; c Department of Cardiovascular Surgery, Valais Hospital, Sion, Switzerland; d Department of Cardiovascular Surgery, Vaud University Hospital, Lausanne, Switzerland

**Keywords:** coarctation of the aorta, muscle-sparing approach, children

## Abstract

**Objective:**

Surgery for aortic coarctation repair provides excellent hemodynamic results but may be complicated by musculoskeletal issues. The purpose of the study was to determine the midterm results of a muscle-sparing surgical approach to aortic coarctation repair, with special emphasis on the repair and on the musculoskeletal changes associated with a posterior thoracotomy.

**Methods:**

We included all children with aortic coarctation operated on with our minimally invasive approach between June 2002 and October 2004, with a follow-up of ≥4.5 years. Patients were assessed clinically and echocardiographically. The spine, left chest, and shoulder were assessed clinically and radiographically.

**Results:**

Thirty-one children were included. The age at operation ranged from 1 day to 15 months and weight ranged from 980 g to 10 kg. All patients underwent an extended end-to-end anastomosis coarctation repair through a minimal (n = 19) or total-muscle sparing (n = 12) or extrapleural (n = 18) approach. Five patients had an additional enlargement procedure on the aortic arch. 27 patients had no residual or recurrent gradient. Four patients exhibited restenosis, for which 1 underwent a percutaneous angioplasty and 2 underwent surgical reintervention. All patients were free of hypertension. One patient had borderline values. The musculoskeletal assessment was normal in all but 3 patients. Two patients who underwent other subsequent thoracic surgeries developed thoracogenic scoliosis of moderate severity. A third patient had a left winged scapula. No rib fusion or intercostal space enlargement was found.

**Conclusions:**

Compared with a conventional approach, our minimally invasive surgical approach led to excellent musculoskeletal outcomes without compromising the hemodynamic results.

**Figure:**
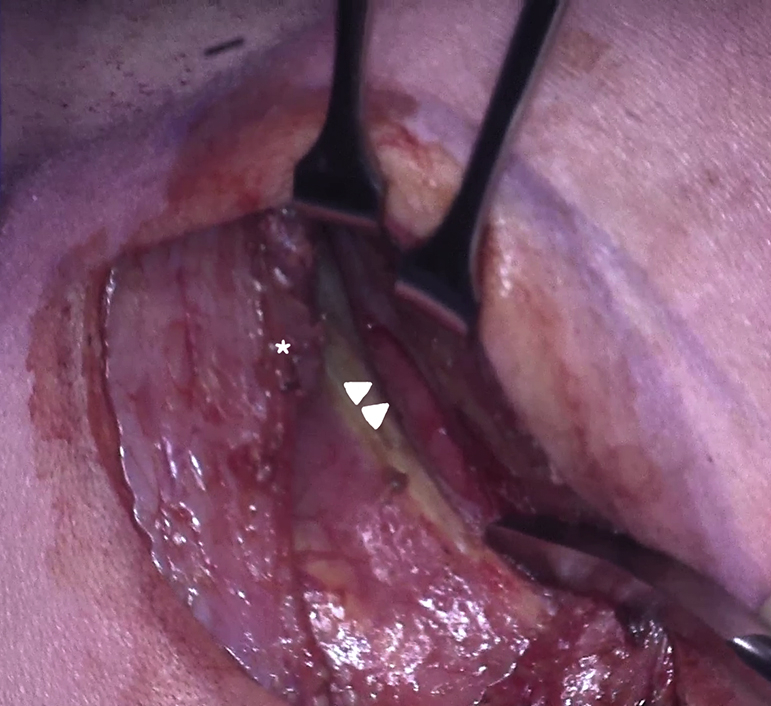
The latissimus dorsi (∗) has been mobilized and preserved, and the tip of the scapula is retracted cephalad. The intercostal space is entered by separating the periosteum is separated from the superior border of the fifth rib (*white arrows*) without dividing any intercostal muscles.

**Central Message**

Compared with a conventional approach, our minimally invasive approach, which respects the chest wall muscles, results in excellent musculoskeletal outcomes without compromising the hemodynamic results of coarctation repair.

**Perspective**

The goal of surgical treatment of aortic coarctation is to relieve the pressure gradient and allow for subsequent growth. Coarctation resection and extended end-to-end anastomosis has become the surgical gold standard. Early and long-term results are excellent. Minimizing the trauma of surgery through a less invasive approach, allows quicker post-operative recovery. This can be achieved by avoiding division of any muscles and by entering the chest with a subperiosteal and extrapleural route.

See Commentary on page 257.

The goal of surgical treatment of aortic coarctation is to relieve the pressure gradient and allow for subsequent growth. Coarctation resection and extended end-to-end anastomosis has become the surgical gold standard. Early and long-term results are excellent. Minimizing the trauma of surgery through a less invasive approach allows for quicker postoperative recovery. This can be achieved by avoiding the division of any muscles and by entering the chest via a subperiosteal and extrapleural route.

In 2002, we introduced a total muscle-sparing surgical approach to minimize the trauma of surgery, and have previously reported our initial experience and hemodynamic results.^1^ Although minimizing trauma is important, priority is given to achieving a flawless repair without residual gradient. Fewer studies have investigated the musculoskeletal outcomes after coarctation repair. Conventional posterior thoracotomy, the most commonly used approach to coarctation, leads to a high rate of postoperative winged scapula (in up to 77% of patients) or scoliosis (up to 46% of patients).^2^, ^3^, ^4^, ^5^

The goal of this study was to determine the midterm results of our total muscle-sparing and extrapleural approach to coarctation, with a special emphasis on the aortic repair and on the musculoskeletal changes linked to the posterior thoracotomy. Our hypothesis was that this minimally invasive approach performed at a young age, while ensuring an excellent repair, could lead to better musculoskeletal outcomes compared with those published in the literature.

## Methods

### Patient Inclusion

We performed a retrospective review of all consecutive neonates, infants, and children who underwent with a muscle-sparing aortic coarctation repair by a single operator between June 2002 and October 2004. We chose this cohort to ensure a minimum 4.5-year follow-up to assess musculoskeletal outcomes. This cohort was previously reported in our description of the technique and initial hemodynamic results.^1^ The exclusion criterion was a follow-up <4.5 years.

Our research plan was accepted by the Swiss Ethics committee (protocol no. 2016-00713). The Swiss Ethics Committee approved that this study be carried out without consent forms thanks to article 34 of the Swiss federal law on human research. Regarding the pictures and the video of the patient, a proper consent form was obtained from the legal representative.

### Surgical Technique

**Figure 1:**
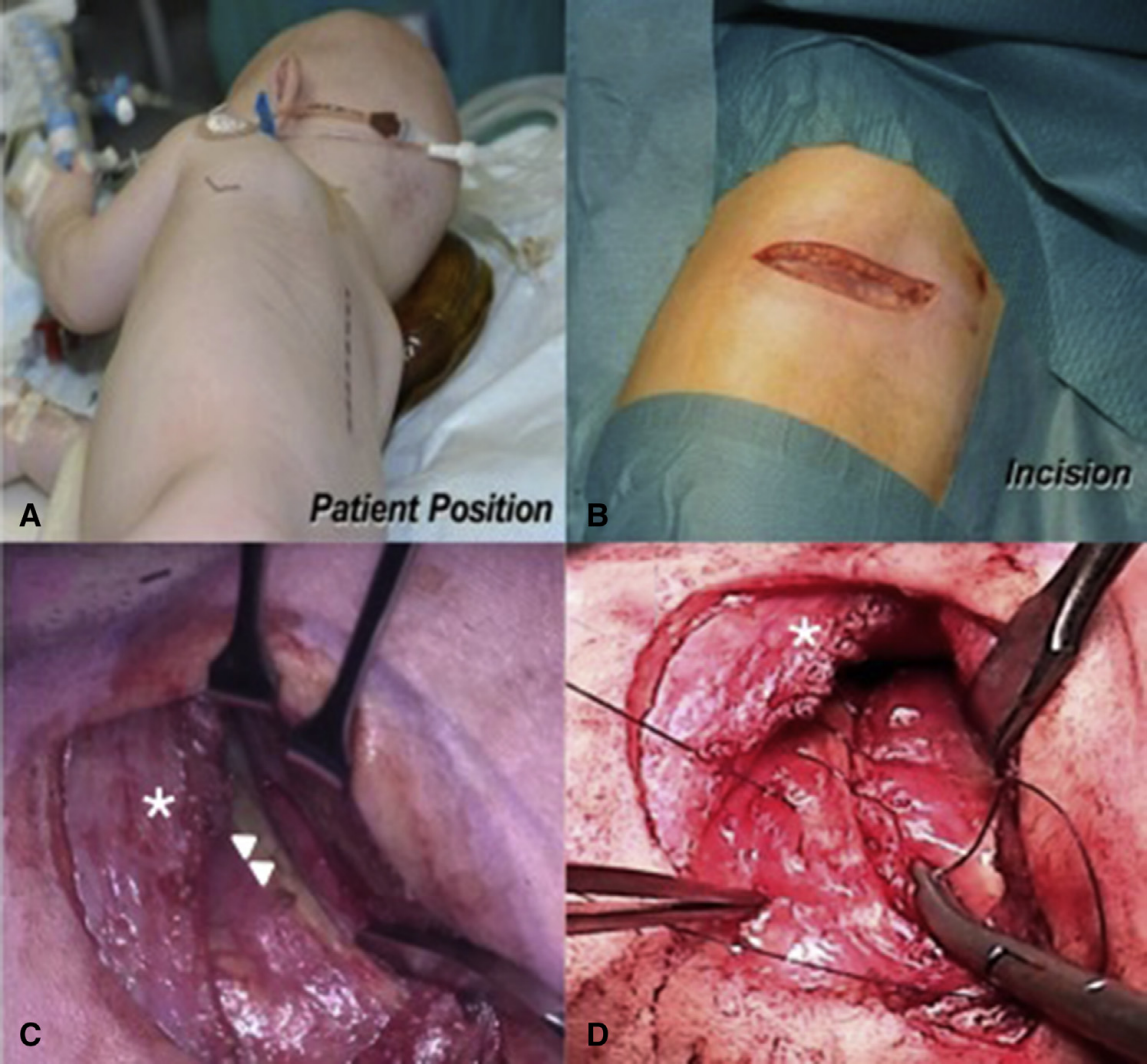
A, Position of the patient before surgery. The *dashed line* marks the spine. The *solid line* shows the tip of the scapula. B, Subscapular incision of 5 cm, 1 cm under the medial border of the scapula. C, The latissimus dorsi (∗) has been mobilized and preserved, and the tip of the scapula is retracted cephalad. The intercostal space is entered by separating the periosteum is separated from the superior border of the fifth rib (*white arrows*) without dividing any intercostal muscles. D, Reconstruction of the intercostal space. The periostium is sutured using a running suture to the rib.

**Figure 2:**
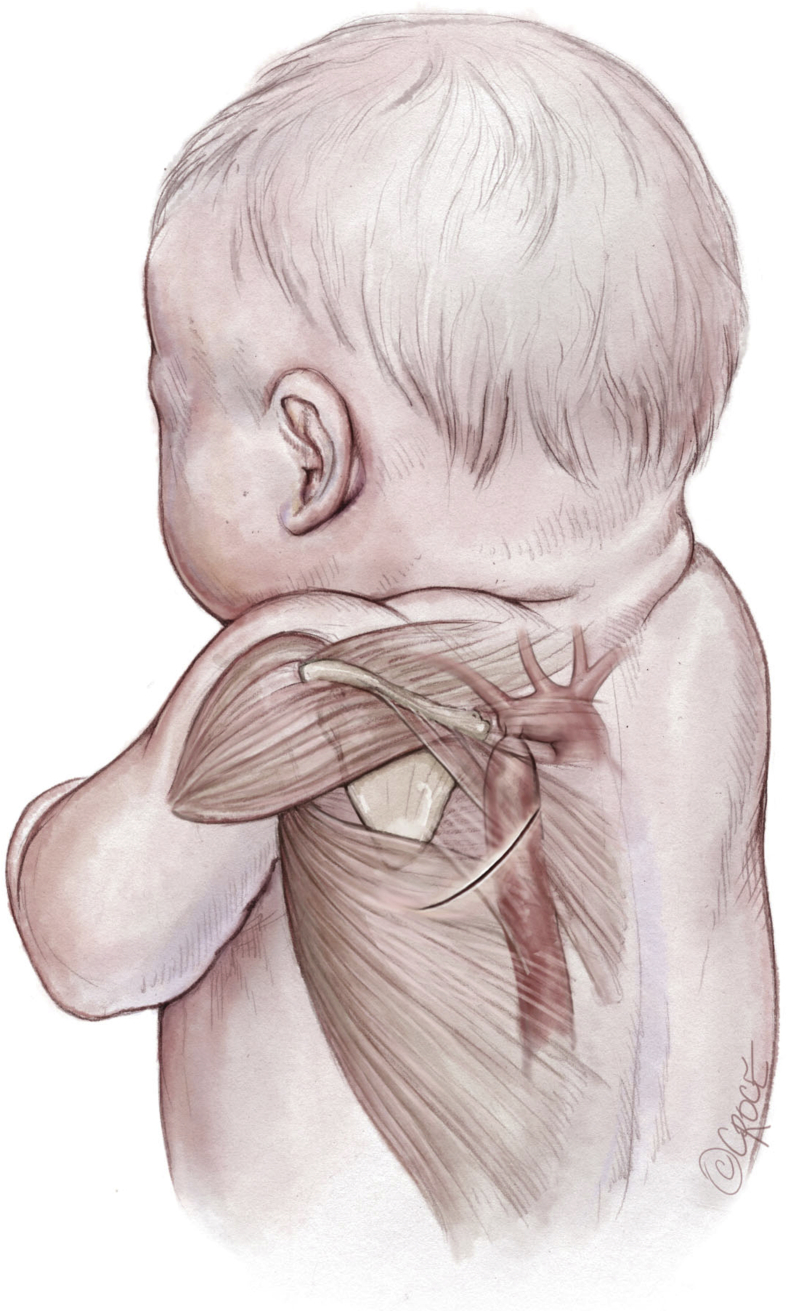
The position of the coarctation of the aorta in the chest. The *solid line* under the scapula shows the incision location.

The surgical technique has been described in detail previously.^6^ The patient is positioned in a right lateral decubitus, turned slightly forward (Figures 1, *A*, and 2). The left arm is rotated forward and upward. The incision is made on the skin facing the so-called “triangle of auscultation,” beginning 1 to 2 cm under the inferior angle of the scapula and directed posteriorly toward the spine (Figure 1, *B*). The length of the incision is related to the size of the patient, but in our series of neonates and small infants it never exceeded 5 to 6 cm. The subcutaneous tissue is undermined over an extensive area to allow visualization of the spinal insertion of the latissimus dorsi. At the beginning of our experience, the posterior part of the latissimus dorsi was divided over several centimeters. With further undermining of the subcutaneous tissue posteriorly, the entire muscle could be preserved, and this has been our approach ever since. The border of this muscle is detached from its aponeurosis for 6 to 7 cm, up to the tip of the scapula. One right-angle retractor is inserted below the latissimus dorsi and the serratus anterior, and another is inserted below the scapula. The loose tissue between the thoracic wall and the muscles is freed, allowing better visualization of the fifth intercostal space (Figure 1, *C*).

The intercostal muscles are preserved by peeling the periosteum from the upper side of the fifth rib, by opening the periosteum with low-energy cautery along the superior border of the rib as far as possible anteriorly. The erector spinae muscles are preserved posteriorly. The periosteum is separated from the rib with a periosteum elevator. The parietal pleura is freed from the thoracic wall with 2 peanut sponges. This maneuver is extended laterally down to the aorta and superiorly to the aortic arch. A small retractor is introduced and opened progressively. Further freeing of the periosteum and of the pleura is performed if necessary to avoid excessive tension on the ribs. The pleura, and the left lung indirectly, are retracted medially with several 6/0 polypropylene stitches inserted in the pleura, close to the aorta. This exposes the thoracic aorta without the need to introduce a classical retractor, which frees the hand of the assistant. The aortic arch and the arch vessels are dissected completely free. The descending aorta and the thoracic arteries are also dissected free as low as possible. The first 2 pairs of intercostal arteries are mobilized over their entire length, to allow sufficient mobilization of the descending thoracic aorta. They are clipped, because the distal clamp will be placed below their level, but not divided, because they usually can be preserved. The clips are removed at the end of the coarctation repair.

**Figure 3:**
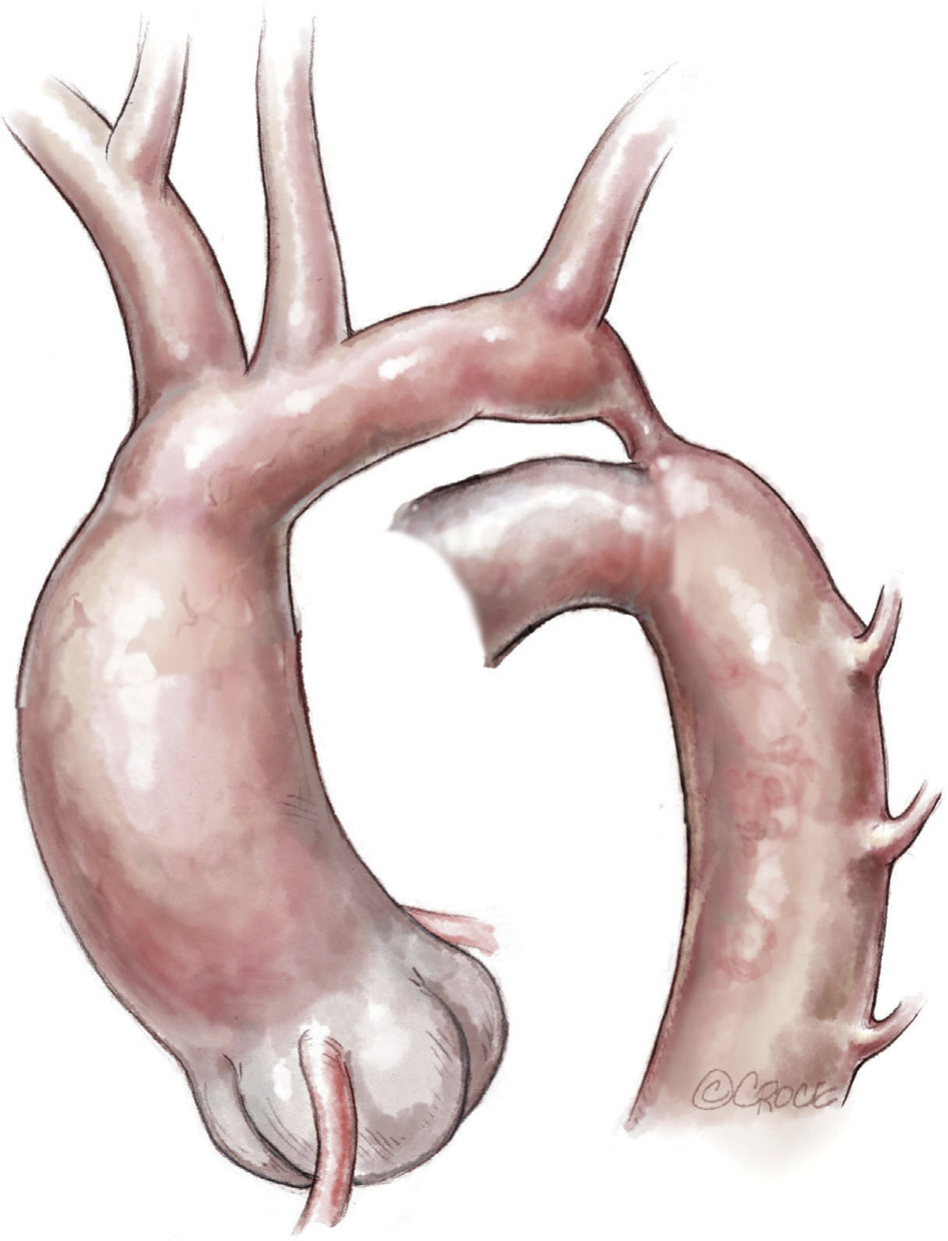
Neonatal ductus-dependent coarctation of the aorta with an elongated and hypoplastic distal arch (between the left common carotid and the left subclavian arteries), a stenotic isthmus, and a ductus arteriosus extending into the descending aorta.

**Figure 4:**
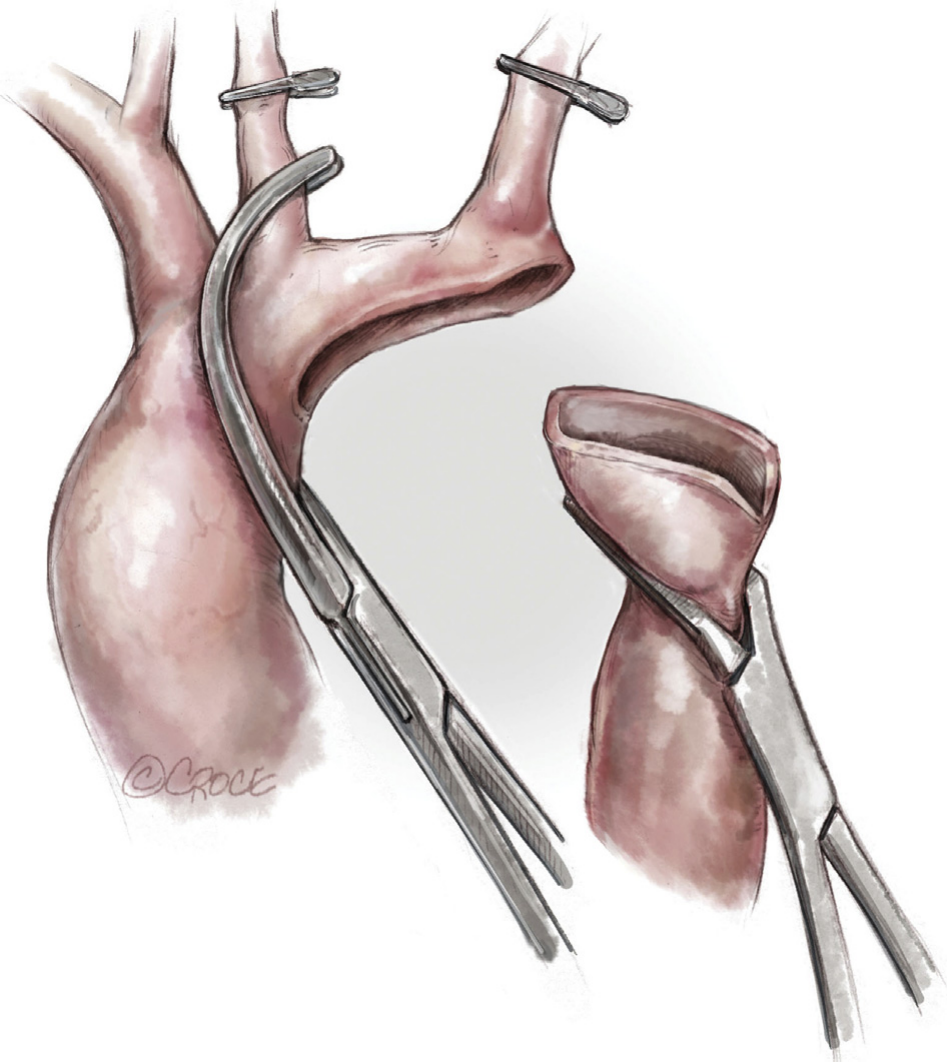
Clamping of the aortic arch between the brachiocephalic trunk and the common left carotid artery. The left subclavian and the left common carotid arteries are clamped by hand-held clips. The ductus arteriosus is ligated and divided. The coarctation is resected. The underside of the aortic arch is fileted open, and a counterincision is made in the opposing proximal descending aorta to allow an extended end-to-end anastomosis.

**Figure 5:**
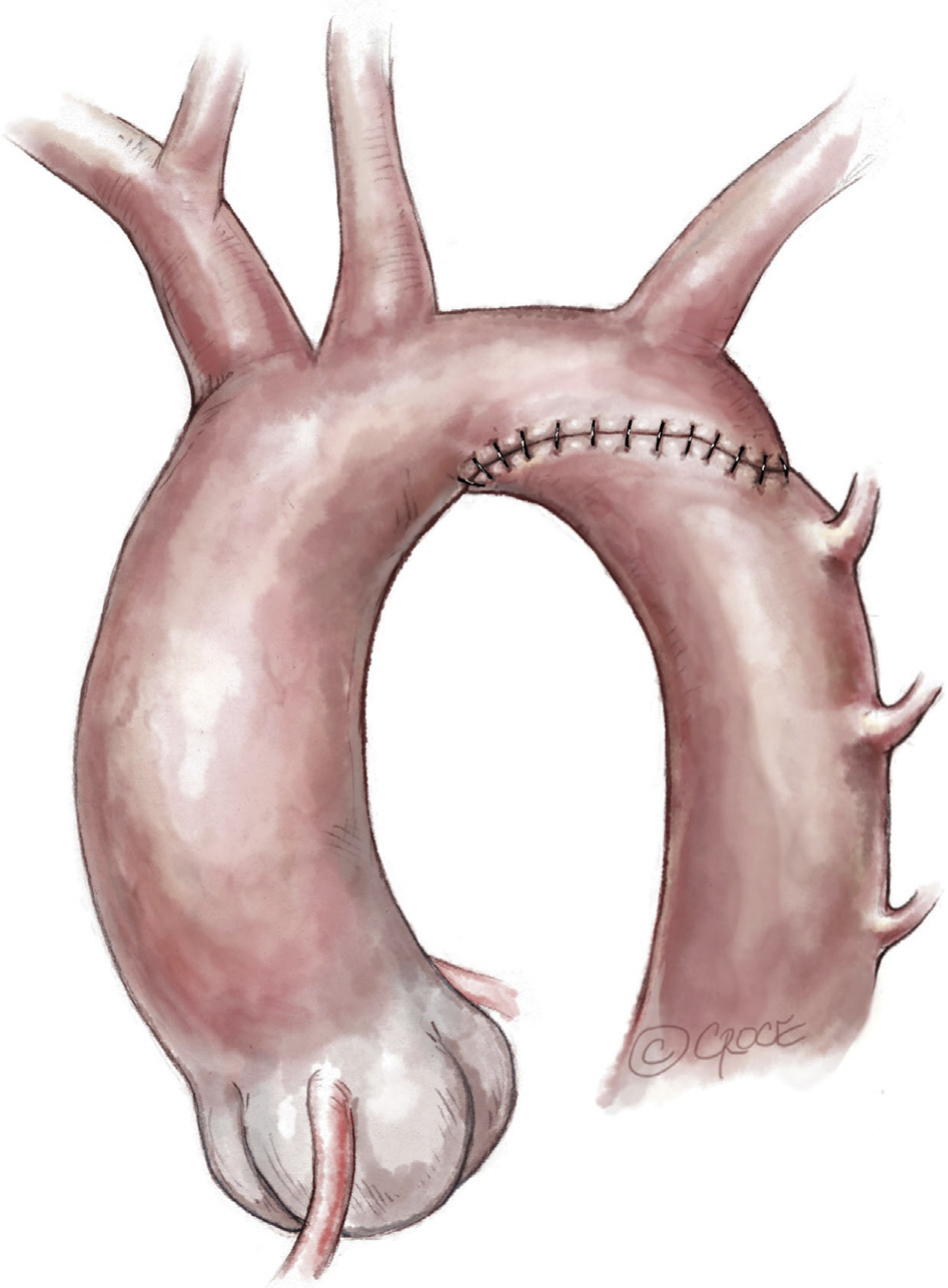
The distal aortic arch and the proximal descending aorta are anastomosed with a running 7-0 or 6-0 polydioxanone suture.

After administration of heparin, the aortic arch is clamped between the brachiocephalic trunk and the common carotid artery, as is the descending aorta. The left carotid artery, left subclavian artery, and first 2 pairs of thoracic arteries were temporarily softly occluded with a hand-held clipper (Figures 3 and 4). The ductus arteriosus is ligated and divided. The coarctation of the aorta is resected. The aortic arch is opened inferiorly up to the proximal clamp, below the takeoff of the left common carotid artery. The descending aorta is shaped to fit this long opening. The distal aortic arch and the proximal descending aorta are sewn together with a running 7-0 or 6-0 polydioxanone suture (Figure 5).

In patients with pronounced hypoplasia of the aortic arch, the arch is enlarged superiorly with a patch of autologous pericardium, while the lower body is perfused by the ductus arteriosus. The coarctation resection and a direct aortic anastomosis are performed after the arch plasty.^7^

Air is removed from the aorta, and the clamps (including the clips on the left subclavian, left common carotid and the intercostal arteries) are removed. A low-vacuum drain is placed in the extrapleural space. The chest wall is reconstructed by reapproximating the peeled-off periosteum onto the bare rib bed using a running polyglactin 2-0 or 3-0 suture. We have never encountered the rib vessels, which run in a groove. This method of closure ensures preservation of the normal rib interspace, because almost no muscle is taken in the bites. The loose tissue below the muscle plane is closed with a fine Vicryl suture. The latissimus dorsi is approximated to its aponeurosis (Figure 1, *D*). A 2-layer absorbable suture closes the skin (Video 1).

### Follow-up and Clinical Assessment

Patients were followed-up through their referring cardiologist and by reviewing their last report. Patients followed for <4.5 years were excluded.

The quality of the aortic repair was assessed by coarctation recurrence and persistent hypertension. Recurrence was defined as a pressure gradient of ≥20 mm Hg across the aortic repair or the need for an endovascular or surgical reintervention. Blood pressure >95th percentile or >140/90 mm Hg was considered hypertension. Both upper and lower limb blood pressure were measured by standard pressure cuffs at the practitioner's office. In borderline patients, 24-hour ambulatory blood pressure was measured.

The status of the left chest and shoulder was assessed clinically and radiographically. The symmetry of the thorax and shoulder was evaluated, with a special emphasis on the recognition of scoliosis and abnormal position of the scapula. Rib fusion or excessive width of the intercostal space was considered a failure. Whenever an anomaly was suspected, a more complete examination was performed by an orthopedic surgeon.

This study relied on descriptive statistics using Excel (Microsoft, Redmond, Wash). Kaplan–Meier estimates were calculated using SPSS version 25 (IBM, Armonk, NY) and GraphPad Prism (GraphPad Software, La Jolla, Calif).

**Figure 6:**
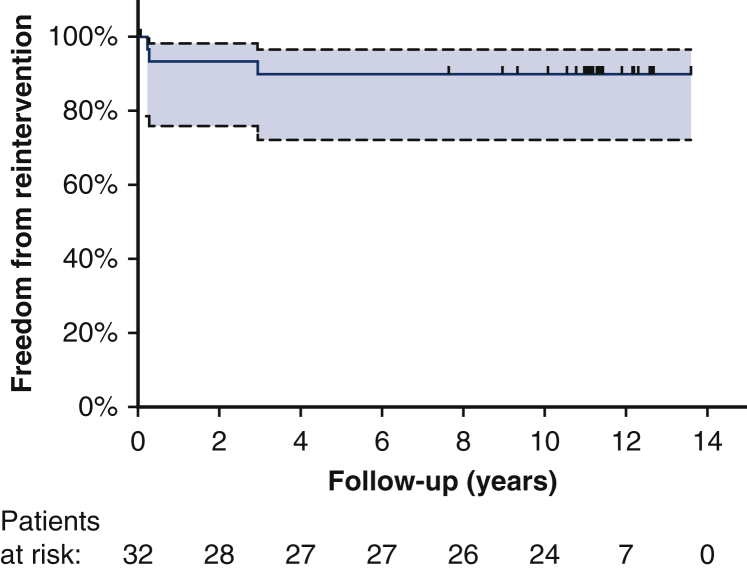
Kaplan–Meier estimates of freedom from reintervention up to 14 years.

## Results

### Patient Population

Forty patients underwent coarctation repair during the study period. Nine patients were excluded due to follow-up below our prespecified minimum (<4.5 years) for an adequate musculoskeletal assessment. Four of these patients had insufficient follow-up, and 5 died from associated diseases, either cardiac (ie, Shone's complex, endocardial fibroelastosis, or intractable pulmonary hypertension) or noncardiac (ie, diaphragmatic hernia or hypoplasia of the right lung). Thus, a total of 31 patients were included in the study; the baseline characteristics of these patients are summarized in Table 1.

**Table 1:** Baseline patient characteristics

Characteristic	Value
Sex, female/male, n	15/16
Age at operation, d, median (IQR)	9 d (5-30, 1-447)
Weight at operation, median (IQR)	3480 g (2900-3910 g, 980 g-10 kg)
Duct-dependency, n (%)	25 (81)
Associated disease, n (%)	30 (97)
Previous balloon angioplasty, n (%)∗	3 (10)

*IQR*, Interquartile range.

∗ The coarctation was slightly dilated to reach a concomitant aortic valve stenosis to dilate the aortic valve. The surgical repair of the coarctation was performed within 2 weeks.

### Surgical Technique

All patients underwent an extended end-to-end anastomosis; 5 patients required an additional enlargement plasty of the aortic arch. In 19 patients, surgical access to the aorta included a minimally invasive posterior thoracotomy with division of the posterior part of the latissimus dorsi for 2 to 3 cm. A total muscle-sparing posterior thoracotomy was performed in 12 patients. In all patients, the intercostal space and muscles were preserved by the subperiosteal access. In 18 patients, the approach was extrapleural.^1^ Operative data and concomitant procedures are summarized in Table 2.

**Table 2:** Operative data

Parameter	Value
Operative time, min, median (IQR)	90 (80-120)
Cross-clamp time, min, median (IQR)	22 (20-29.25)
Procedure, n	
Extended end-to-end anastomosis	31
Pulmonary artery banding	5
Enlargement-plasty of the distal arch	4
Subclavian retrograde flap plasty	1

*IQR*, Interquartile range.

### Follow-Up

During a mean follow-up of 11.2 ± 1.5 years, 1 patient died at age 11 years of pulmonary hypertension after repeated bronchoaspiration and in a context of multiple contraindications for lung transplantation. This patient had never needed a reintervention regarding the coarctation.

### Hemodynamic Outcomes

Twenty-seven patients showed an excellent result, with no residual or recurrent stenosis (87.1%).^5^ Four patients showed a residual gradient (>20 mm Hg at rest), for which 1 patient underwent balloon angioplasty and 2 patients underwent a second surgical repair (Figure 6).

Patient A required balloon angioplasty because of a 26-mm Hg peak gradient across the isthmus and stenosis of the left subclavian artery origin. Patient B presented with a 25-mm Hg gradient across the proximal aortic arch between the brachiocephalic artery and the left common carotid artery; this was repaired surgically by patch enlargement of the inferior part of the arch through a sternotomy and using cardiopulmonary bypass.^8^ The patient showed a subsequent wide aortic arch without any gradient across the arch or the isthmus. Patient C underwent a surgical reintervention because of a 27-mm Hg gradient across the distal aortic arch. Reoperation consisted of a superior arch enlargement with a subclavian artery flap (a reverse Waldhausen procedure) and reimplantation of the distal left subclavian artery in the left carotid artery, at the thoracic outlet. Patient D showed a stable 21 mm Hg gradient across the aortic arch and was not reoperated on. Transthoracic echocardiography showed a good repair result at 10 years and a mild turbulent flow acceleration just after the left subclavian artery takeoff that had been stable over the years, with no hemodynamic consequences. This patient had normal blood pressure values.

In terms of blood pressure, all patients showed normal values except patient C, who showed a tendency to have values >95th percentile. No patient was receiving antihypertensive medication.

### Musculoskeletal Outcomes

All the patients for whom detailed orthopedic data were obtained (n = 25) had good mobility of the back and left shoulder at midterm follow-up, with the exception of 1 patient, in whom this was not reported. The alignment of the thoracolumbar spinous processes was within the normal range in all but 2 patients (7.4%). One patient had a lumbar (L2) left-convex scoliosis of 25° with a thoracic (T8) right-convex scoliosis of 28° and pectus excavatum at 12.5 years. This patient, who presented with a double-outlet right ventricle, had undergone a posterolateral thoracotomy using a partial muscle-sparing thoracotomy approach with division of the posterior part of the latissimus dorsi over several centimeters. The operation included coarctation of the aorta repair and a pulmonary artery banding at age 2 days and a sternotomy 4 months later for the removal of a pulmonary artery band and closure of a ventricular septal defect and an atrial septal defect. The other patient had a right-convex thoracolumbar scoliosis of 24° at 9.5 years. Within the first 2 days of life, he had undergone a right lateral thoracotomy (without muscle preservation) to correct esophageal atresia and a left posterior thoracotomy using a total muscle-sparing and extrapleural approach for the coarctation repair. The correction of the aorta included a subclavian flap plasty and reimplantation of the left subclavian artery onto the left common carotid.

Twenty-eight patients had normal shoulder motion and a normal shoulder appearance. One patient had a left winged scapula (3.6%) with normal shoulder motion. He had undergone repair of coarctation of the aorta with left subclavian artery translocation, using a partial muscle-sparing approach, at 8 days of age and was the patient who required balloon angioplasty 3 months later for restenosis.

All chest radiographs showed normal rib cage anatomy, without any rib fusion or excessive enlargement of the intercostal space.

## Discussion

With increasingly less-invasive repairs and progress in interventional cardiology, our goal was to determine the benefits of a minimally invasive surgical approach, focusing on the midterm results with special emphasis on the surgical repair of the aorta and on the musculoskeletal changes associated with posterior thoracotomy. Our minimally invasive approach showed excellent hemodynamic results and a superior outcome in terms of shoulder and back appearance and mobility compared with a conventional approach, as summarized in Table 3.

**Table 3:** Comparison of studies

Study	Patients, n	Age at operation, y, mean ± SD	Follow-up, y, mean ± SD	Muscle-sparing PLT	Scapula alata, %	Scoliosis, %
Present study	31	0.1 ± 0.2∗	11.2 ± 1.5	Yes	3.6†	7.4‡
Bal et al, 2003^2^	49 CHD	3.8 ± 4	6	No	77	31 (3/5 with CoA)
Emmel et al, 1996^3^	21 CoA	<1	≥9	No	57.1	NA
Roclawski et al, 2012^4^	45 CoA	6.9	14.8	No	NA	46.6
Van Biezen et al, 1993^5^	160 CoA		12	No	NA	22
Kucukarslan et al, 2006^16^	90 non-CHD	4.2 ± 2.91	5.65 ± 2.83	Yes (n = 40)	12.5 (n = 5)	2.5 (n = 1)

*SD*, Standard deviation; *PLT*, posterolateral thoracotomy; *CHD*, congenital heart defect; *CoA*, coarctation of the aorta; *NA*, not available.

∗ For the purpose of comparison, we used the average, although the median, as in Table 1, would be more appropriate in describing our cohort.

† n/N = 1/28.

‡ n/N = 2/27.

### Quality of the Aortic Repair

Ensuring the quality of aortic repair is our central concern. Hypertension is one of the major predictors of long-term survival.^9^^,^^10^ It may occur in up to 45% of patients after surgical repair, although surgery during infancy reduces the risk down to approximately 10%. Our minimally invasive approach shows results comparable to those from a conventional approach. In our cohort, only 1 patient (3.2%) showed a trend toward hypertension >95th percentile, and no patient required any antihypertensive treatment. Our restenosis rate of 12.9% is comparable to rates reported by other groups performing this correction in the neonatal period.^11^, ^12^, ^13^ Of note, in 3 of the 4 patients with recurrent obstruction, the narrow part of the aorta was in the proximal aortic arch (between the brachiocephalic artery and the left common carotid artery), which could be relieved in 2 patients with an arch plasty through a sternotomy. In 1 patient, stenosis recurred at the level of the repair and necessitated balloon dilatation. Overall, our results demonstrate that our minimally invasive approach allowed us to perform an extended resection of the coarctation of the aorta in all patients, with the same outcome.

### Musculoskeletal Outcome

Depending on the authors and on the method of diagnosis, the estimated prevalence of scoliosis in the population presenting with congenital cardiac diseases, is reported to range between 0.2% and 31%, compared with a rate of 2% or 3% in the general population.^2^^,^^4^^,^^5^^,^^14^, ^15^, ^16^ Similarly, Reckles and colleagues^17^ found that among a cohort of patients with scoliosis, 5% had a cardiac congenital defect, a 10-fold higher rate than that in the general population. Although the relationship is still unclear, some major etiologic factors include the variety of cardiac congenital diseases, genetic predisposition, and the surgical approach possibly leading to thoracogenic scoliosis.^2^^,^^4^ Other studies showed that patients with cyanotic cardiac disorders tended to show a higher prevalence of scoliosis, suggesting that hypoxia may be involved in its genesis.^3^^,^^4^^,^^17^ Obviously, mechanical causes after surgery (eg, rib resection, rib fusion, muscles division, and consequent nerves injury—anything that weakens and deforms the chest wall) can account for such a subsequent development. According to Van Biezen and colleagues,^5^ eliminating the function of the intercostal muscles has the same effect as a paralysis and weakens the thoracic wall. Finally, Emmel and colleagues^3^ showed that performing thoracotomy at a very young age (under 1 year) increases the risk of subsequent scoliosis. The relatively forceful spreading of the ribs and excessive stress on the costovertebral joints may account for this higher prevalence.

Our 7.4% prevalence of scoliosis compares favorably with any other reported results of posterior thoracotomy and even of angioplasty. Two patients in our cohort developed scoliosis, which does not appear to be related to the incision. One patient with other thoracic musculoskeletal deformities might have developed scoliosis spontaneously, and the other patient developed scoliosis on the right side, which was used by general surgeons to repair a concomitant esophageal atresia. Only a few studies have focused on this midterm complication. They showed how severe the musculoskeletal degradation after a thoracotomy might be (Table 3). In this regard, angioplasty proponents may claim a better musculoskeletal outcome. To illustrate this, Roclawski and colleagues^4^ compared the prevalence of scoliosis in patients treated for coarctation of the aorta surgically or by angioplasty. The difference—46.6% versus 16.6%—was significant. A comparison of the results of that study with ours might be biased, because those studies involved older patients than ours, with an average respective age at intervention of 6.9 and 9.3 years in their 2 groups. In another mixed study, Kucukarslan and colleagues^16^ found a better rates, similar to ours, of 2.5% for scoliosis and 12.5% for scapula alata in 90 patients, 40 of whom had undergone a muscle-sparing approach. In their approach, the serratus anterior was spared but the intercostal muscles or the latissimus dorsi were not, as they were in ours.

Regarding winged scapula, the prevalence oscillates between 12.5% and 77% in published reports (Table 3). Such high numbers result from old approaches, when the thoracotomy incision was especially wide, encompassing half of the left hemithorax. Currently, this prevalence certainly ranges closer to 10% to 15%.^16^ We encountered one such complication, which looked like a hernia of the scapula tip through a weakened muscle layer or through a loose adaptation of the latissimus dorsi muscle to the posterior aponeurosis. Today, we close our parietal muscular plane in 2 layers, fixing the scapula tip to the serrratus anteriosus and the latissimus dorsi again to its aponeurosis. This strengthened closure should prevent any further deformity of the left shoulder.

The majority of the previously reported studies (Table 3) focused on musculoskeletal outcomes after thoracotomy performed at a time when surgeons were using generously large incisions. The amount of muscle severed has been reduced “almost spontaneously” over time in most units, and the contemporary risk of developing a chest wall deformity is certainly lower than the figures reported in these historical series. At times, at the beginning of our experience and in older children, we have partially divided the latissimus dorsi, having always privileged the quality of the aortic repair, without seeing any negative effects on the child's final stature. Nonetheless, any disturbance of the forces applied to the thoracic wall in a growing child can have an impact. We believe that this hazard is minimized with our approach.

### Study Limits and Strengths

This study has several limitations. First, it is a single-center retrospective study of a small sample size without a control group. We did not have a concomitant or historical control group that could be used for comparison and relied on comparisons with published reports, of which some were performed in earlier periods using techniques that were less respectful of tissue. Finally, the definition of scoliosis and winged scapula may have differed among studies, making the comparatives figures less robust. Nonetheless, we tried to include all the musculoskeletal complications in this study by selecting patients who had at least 4.5 years of follow-up, because scoliotic problems seldom develop before age 3 years.^5^

In conclusion, in our cohort, our minimally invasive approach that respects the chest wall muscles resulted in excellent musculoskeletal outcomes without compromising the hemodynamic results of coarctation repair.

### Conflict of Interest Statement

The authors reported no conflicts of interest.

The *Journal* policy requires editors and reviewers to disclose conflicts of interest and to decline handling or reviewing manuscripts for which they may have a conflict of interest. The editors and reviewers of this article have no conflicts of interest.

## Supplementary Data

Video 1 Our technique of muscle-sparing coarctation repair. Video available at: https://www.jtcvs.org/article/S2666-2507(20)30226-1/fulltext.
