# Supplementary figures and images for: Muscle-sparing aortic coarctation repair
Source: JTCVS Tech. 2020 May 17;3:249–56. doi: 10.1016/j.xjtc.2020.05.005 (PMC8302918; doi:10.1016/j.xjtc.2020.05.005)

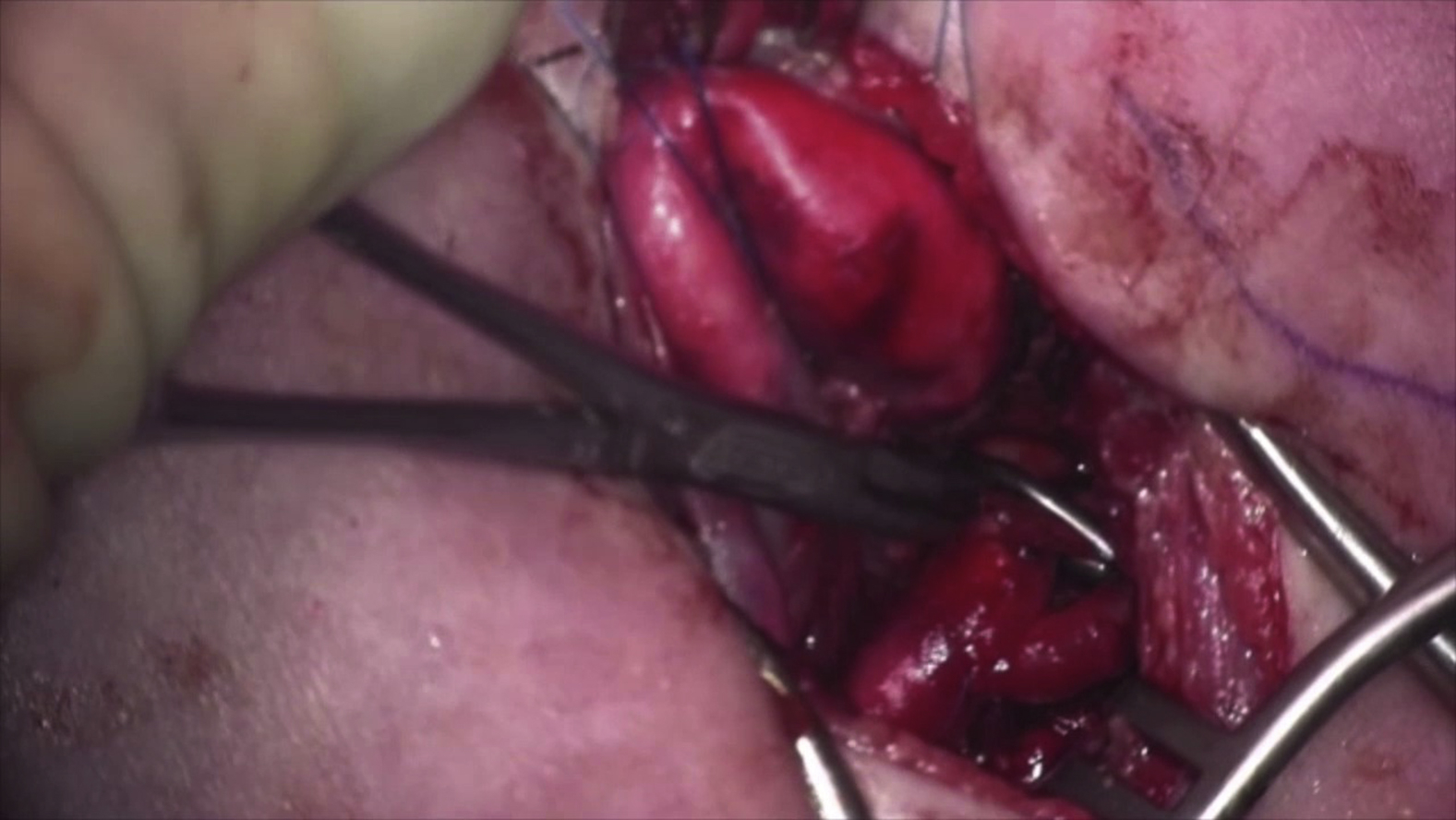

Supplement: Video 1 — Our technique of muscle-sparing coarctation repair. Video available at: https://www.jtcvs.org/article/S2666-2507(20)30226-1/fulltext. [file fx2.jpg]
